# What are the neural correlates of dissociative amnesia? A systematic review of the functional neuroimaging literature

**DOI:** 10.3389/fpsyt.2023.1092826

**Published:** 2023-01-26

**Authors:** Simon Taïb, Antoine Yrondi, Béatrice Lemesle, Patrice Péran, Jérémie Pariente

**Affiliations:** ^1^INSERM U1214 Centre d’Imagerie Neuro Toulouse (ToNIC), Toulouse, France; ^2^Service de Psychiatrie, Psychothérapie et Art-Thérapie, Centre Expert du Stress Traumatique, CHU de Toulouse, Toulouse, France; ^3^Service Universitaire de Psychiatrie et Psychologie Médicale, CHU de Toulouse, Toulouse, France; ^4^Pôle Neurosciences, CHU de Toulouse, Toulouse, France

**Keywords:** cognitive disorder, memory loss, hysteria, dissociative disorder, stress, trauma, MRI, PET

## Abstract

**Aim:**

Dissociative amnesia is an emblematic psychiatric condition in which patients experience massive memory loss ranging from focal to global amnesia. This condition remains poorly understood and this review aims to investigate the neuroanatomical feature of this disease.

**Methods:**

We conducted a systematic review of the scientific literature available on PubMed, up to December 1, 2022, using a combination of keywords referring to dissociative amnesia. We included every scientific report involving patients undergoing a functional imaging procedure.

**Results:**

Twenty-two studies met our inclusion criteria (gathering 49 patients). Only one was a controlled study with a large sample. The other 21 were case reports and case series. In resting state, neuroimaging studies mostly showed a hypo-activated right inferolateral prefrontal cortex, associated with limbic hypoactivity and lesser activation of the hippocampal and para-hippocampal structures. The patients also presented abnormal patterns of cerebral activation when performing memory tasks. When testing recognition of memories from the amnestic period, patients showed increased activation across temporal areas (hippocampal and para-hippocampal gyri) and the limbic network. When trying to recollect memories from an amnestic period compared to a non-amnestic period, patients failed to activate these structures efficiently. Most of these patterns tended to return to normal when symptoms resolved.

**Conclusion:**

This review identified a paucity of controlled studies in the field of dissociative amnesia neuroimaging, which restricts the extrapolation of results. Patients with dissociative amnesia present a broad prefronto-temporo-limbic network dysfunction. Some of the brain areas implicated in this network might represent potential targets for innovative treatments.

## 1. Introduction

Dissociative amnesia (DA) is a psychiatric disease in which patients experience a failure to recall important autobiographical memories in the absence of any known neurological or other medical condition (1). Due to the imprecision of the psychiatric definition, DA encompasses a broad group of clinical presentations (2). Some patients experience a focal loss of memory encompassing a limited time-period (e.g., 5 years of their life) or a specific theme (such as a relationship or a work experience), most of the time stressful. However, some patients exhibit a more spectacular display of their symptoms with a global amnesia encompassing their whole lifespan and even sometimes a loss of identity and a fugue.

Dissociative amnesia often appears after mild traumatic brain injuries (3) or psychological stressors (4). Its prevalence rates are estimated between 0.4 and 7.3%, with a sex ratio of 1:1 (5). It occurs mostly between the age of 20 and 40 years old, even though cases among children and older people have been described (5). Its pathophysiology has not been elucidated yet. Nonetheless, two theories have arisen in the past decades. Kopelman’s theory of the “stress-related explanatory model” (6, 7) stated that patients suffering from DA were more likely to have prior biopsychosocial vulnerability, with a weaker cognitive executive reserve, leading to an inability to recall old memories. According to this theoretical model, by acting on executive functioning, stress may weaken the ability to retrieve their autobiographical memories (8). More recently, Markowitsch and his colleagues hypothesized that the memory recollection being blocked was under the influence of a fronto-temporal desynchronization due to a dysfunction in the hypothalamic-pituitary-adrenal (HPA) axis (9). The second pathophysiological lead (namely the “two-hit hypothesis”), which does not exclude the first theory, assumes that patients with DA suffer from an additive interaction between both physical and psychological factors causing the amnesic state (10). To date, its treatment relies on the management of the potential underlying depressive disorder, the identification and resolution of the eventual underlying crisis and a contextual interview of memories progressively moving from earlier and less stressful events to more recent ones (2). Contemporary psychotherapeutic techniques include cognitive behavioral therapy and acceptance/commitment therapy (11).

To our knowledge, no previous review has focused specifically on the functional brain alterations observed when using neuroimaging in patients with DA. In the present systematic review, we gathered and analyzed the data available to draw future perspectives in the field of neuroimaging studies and in the treatment of DA.

## 2. Methods

We conducted a systematic review of the international scientific literature using the bibliographic search engine PubMed. The following medical subject headings were used, accordingly to the possible denominations of DA developed in Staniloiu’s review article (5): (“hysterical amnesia” OR “dissociative amnesia” OR “dissociative fugue” OR “psychogenic amnesia” OR “functional amnesia” OR “mnestic block syndrome” OR “medically unexplained amnesia” OR “idiopathic amnesia” OR “disproportionate persistent retrograde amnesia” OR “fugue” OR “focal retrograde amnesia”). These were combined with keywords related to functional neuroimaging (“neuroimag*” OR “MRI” OR “fMRI” OR “PET” OR “SPECT” OR “imaging”).

Our inclusion criteria were the following: studies published up to December 1, 2022, involving patients with DA according to the *Diagnostic and Statistical Manual of Mental Disorders* (fifth edition or prior) who had undergone at least one brain imaging procedure (regardless of the technique used) while they were amnestic.

We searched the database using a predefined search algorithm to identify potentially eligible studies. Articles in which patients had isolated anterograde amnesia with no retrograde amnesia were withdrawn because of the probable different brain mechanisms implicated in their pathophysiology (12). We discarded studies involving EEG investigation because of the poor spatial resolution of this technique. Studies only involving structural techniques were also put aside as they did not add any functional information. All duplicate studies were removed. We independently selected studies based on their titles. Then, all online abstracts were reviewed, and full-text papers were retrieved when relevant. The procedure followed the Preferred Reporting Items for Systematic Reviews and Meta-Analysis criteria (PRISMA) (13).

### 2.1. Quality assessment of studies

The overall quality assessment of each study included in the review was evaluated as follows. We based the ratings on the proportion of «yes» responses to the following criteria; 1/corrected for healthy controls (HC), 2/no prior neurological or psychiatric conditions, 3/absence of active central nervous system condition, 4/absence of active psychiatric condition, 5/effect of medication controlled, 6/diagnosis confirmed by a neurologist and/or a psychiatrist, and 7/sufficient details in the method sections that would allow study replication. Each «yes» added one point to the total score. We added a last item related to the sample size. Studies with 15 patients or more were rewarded 3 points; the ones between 5 and 14 included gave 2 points, and studies with less than 5 patients were only given 1 point. This rating led to an overall score between 1 and 10. When one of the above-mentioned points was not clearly explicated in the study included, we considered that the bias was not considered.

### 2.2. Grouping and task analysis

It has been long known that memory is composed of several systems supported by various anatomical structures (14). Based on this theory, the main symptom in DA (i.e., the loss of autobiographical memory) implies an impaired declarative memory. According to Aggleton and Brown, declarative memory relies on two parallel systems that are recollection and recognition (15). Other systems implicated in declarative memory are the encoding process and the stockage of the memories (16). These systems are under the dependence of numerous brain areas, among which the frontal regions, the medial temporal lobe and the limbic system. To facilitate the understanding and limit the heterogeneity in the review, we grouped some of the studies included into several cluster based either on anatomical features or on the task that the subject was asked to perform.

## 3. Results

**FIGURE 1:**
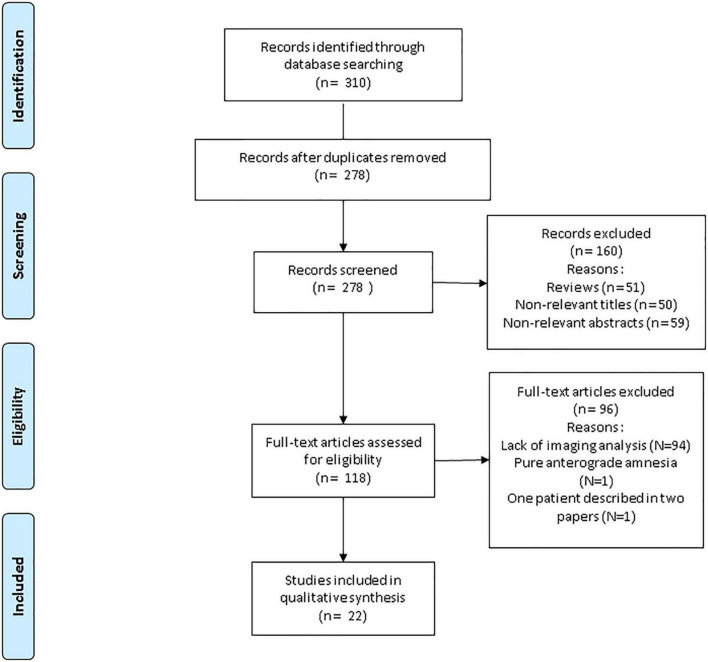
PRISMA flowchart of the systematic review.

We found 310 studies. 32 were duplicates. Among the 278 remaining, 51 were reviews, and 109 were considered irrelevant either because of their title (17) or their abstract (18). Through the 118 last, 94 lacked imaging analysis and one was about a pure anterograde amnesia. Also note that one study was discarded (19) because the subjects it described was presented elsewhere (18). Finally, we identified 22 studies (one large-sample retrospective controlled study and 21 case reports or case series) fulfilling our inclusion criteria, with a total number of 49 individual patients (see Figure 1, PRISMA flowchart of the review) (18, 20–40). Table 1 summarizes the main features (socio-demographic data and clinical features of the patients, experimental paradigms, delay to imagery) and results of the studies included in the review.

**TABLE 1:** Main features of the studies included in the review.

Experimental paradigms			Article	Article type	Imaging technique	Socio-demographical data	Clinical features	Psychiatric and neurological comorbidity	Results (Brodmann area, when available)	Delay to imagery
Pathological condition	Resting-state neuroimaging studies		Brand et al. (18)	Retrospective controlled study,	[^18^FDG]- PET	14 patients (11 men), mean age 36, 6 years old. 19 healthy controlled, paired	Autobiographical retrograde amnesia	Psychiatric and neurological history were exclusion criteria	Right inferolateral prefrontal hypometabolism (BA n.a.)	n.a.
			Hennig-Fast et al. (22)	Case-report	[^18^FDG]- PET	39yo, man	GARA	Previously treated alcohol dependence. Anxious-depressive and narcisistic personality strucutre	Hypometabolism in the right temporo-mesial region	Within first weeks
			Kitamura et al. (23)	Case-report	11C WAY-100635 PET (5-HT 1A receptor)	30yo, man Comparison to 14 healthy controls	GARA	None	No difference at onset	1 month
			Magnin et al. (25)	Case-report	SPECT	51yo, man	GARA, LOI and AA	Functional disorder (abnormal movement and sensitive disorders) Major depressive disorder ongoing since 6 months prior onset of amnesic symptoms.	Hypoperfusion of bilateral temporal lobes, bilateral frontal superior gyri, bilateral orbitofrontal gyri and right head of the caudate nucleus	1 month
			Piolino et al. (27)	Case-report	[^18^FDG]- PET	42yo, man	GARA and LOI	None	Decreased metabolism in the right ventral frontal gyrus (BA 11)	16 months
			Thomas-Antérion et al. (28)	Case-report	[^18^FDG]- PET	30–40yo, man	GARA and LOI	None	Decreased metabolism in the left temporal polar cortex (BA 38), left amygdala, left hippocampus, left para-hippocampus (BA 36), left fusiform gyrus (BA 20 and BA 37) and the left insula.	15 months
			Thomas-Antérion et al. (29)	Case series	[^18^FDG]- PET	Case 2: 19yo man Case 3: 19yo, man	GARA	None for both cases	Decreased right posterior middle temporal gyrus	n.a.
			Tramoni et al. (30)	Case-report	Structural MRI, MTR and spectroscopy	34yo, man 25 healthy controls	GARA and LOI	None	Decrease magnetization transfer ratio and low NAA/(Cho + Cr) ratio in the right prefrontal lobe	n.a.
			Arzy et al. (32)	Case-report	[^18^FDG]- PET	30yo, woman 12 healthy controls	FRA and LOI	None	Increased metabolism of the bilateral posterior parietal cortex (left predominance), and the left inferior frontal cortex. Decreased metabolism of the left inferior temporal cortex.	n.a
Pathological condition	Resting-state neuroimaging studies		Helmes et al. (34)	Case-report	SPECT	54yo, woman	FRA and LOI	Active mild to severe major depressive disorder	Hypoperfusion of the left temporal pole and the left frontal pole, with cerebellar diaschisis	Few weeks (non-precisely specified)
			Markowitsch et al. (26)	Case-report	SPECT	30yo, man	FRA	Possible past meningitis No psychiatry comorbidity	Hypoperfusion of the right inferior frontal cortex and the right anterior temporal cortex	3 weeks
			Mitsui et al. (40)	Case-report	SPECT	40yo, man	GARA	Active mild major depressive disorder	Decreased perfusion of the right ventral area of the medial temporal lobe	2 weeks
			Sellal et al. (37)	Case-report	SPECT	33yo, man	FRA	None	Hypoperfusion of the right anterior temporal regions	Within first days
			Stracciari et al. (38)	Case series	SPECT	Case 1: 20yo, man Case 2: 25yo, woman	FRA in both cases	None	Case 1: left frontal hypoperfusion Case 2: right frontal hypoperfusion 11 other cases with normal SPECT and/or [^18^FDG]- PET	Within first weeks
	Task-performing neuroimaging studies	Retrograde memory (recognition)	Chechko et al. (20)	Case report	fMRI (familiarity task)	41yo, woman	GARA	None	In the familiar condition, higher BOLD signal across the cuneus, the precuneus, bilateral middle occipital gyrus, bilateral middle temporal gyri, bilateral inferior parietal lobule, left fusiform gyrus, left orbitofrontal regions and bilateral amygdala/hippocampi complexes.	Within the first week
			Glisky et al. (21)	Case-report	fMRI (recognition of forgotten native language vs. non-words)	33yo, man	GARA and LOI	None	Decreased BOLD signal in the striatum compared to controls.	Within first months
			Hennig-Fast et al. (22)	Case-report	fMRI	39yo, man	GARA	Previously treated alcohol dependence. Anxious-depressive and narcisistic personality strucutre	Increased BOLD signal across the left anterior cingulate cortex, the left inferiorfrontal gyrus, the left inferior temporal gyrus, the right mesial temporal gyrus, the bilateral inferior occipital gyri, the left lingual gyrus, and the bilateral fusiform gyri.	Within first weeks
			Markowitsch et al. (26)	Case-report	[^15^H_2_0]-PET: 3 conditions [A: resting state; B: when hearing autobiographical sentences from before his fugue (=semantic); C: when	37yo, man	GARA and LOI	None	Decreased rCBF in the right temporal and insular region	8 months
	Task-performing neuroimaging studies	Retrograde memory (recognition)		hearing autobiographical sentences from after his fugue (=episodic)]						
			Yasuno et al. (31)	Case-report	^[15^H_2_O]-PET (recognition of famous faces from amnestic period)	33yo, woman	GARA	None	Increased rCBF in the left anterior cingulate cortex (BA 24 and 32), the caudate, the left putamen, the right medial prefrontal cortex (BA 6 and 8) and the right anterior medial region (including amygdala). Decreased rCBF in the left superior temporal cortex (BA 22 and 42), the right inferior parietal cortex (BA 40), the right anterior cingulate cortex (BA 32) and the right hippocampal region (BA 35 and 36).	2 months
			Arzy et al. (32)	Case-report	fMRI (discrimination of memories from amnestic and non-amnestic periods)	30yo, woman	FRA and LOI	None	Increased BOLD signal of the posterior parietal cortex when seeing pictures from the manestic period	n.a.
			Kikuchi et al. (35)	Case series	Functional MRI (face and name recognition tasks)	Case 1: 27yo, man Case 2: 52yo, man	FRA (both cases)	Case 1: None Case 2: None	Increased BOLD signal of the right dorsolateral prefrontal cortex (BA 9, 10 and 46), the left dorsolateral prefrontal cortex (BA 46), the ventromedial prefrontal cortex (BA 47) and the left ventrolateral prefrontal cortex (BA 47). Decreased BOLD signal of the left hippocampus	n.a.
			Yang et al. (39)	Case-report	Functional MRI (presentation of faces in 3 conditions: faces of people known from amnestic period, faces of people known from preserved period, unknown faces)	22yo, woman	FRA	None	Decreased BOLD signal of the bilateral amygdalae, insulae hippocampi, and para-hippocampal gyri	n.a.
		Retrograde memory (recollection)	Botzung et al. (33)	Case-report	fMRI (memory recollection from amnestic and non-amnestic time periods) using mental imagery	38yo, woman	FRA	None	Decreased BOLD signal of the left parahippocampal gyrus (BA 35 and 36), the left anterior cingulate cortex (BA 24), the left dorsolateral prefrontal cortex (BA 9) and right	Several years
	Task-performing neuroimaging studies								middle lateral frontal gyrus (BA 6). Increased BOLD signal temporo-parieto-occipital loci (BA 7, 13, 18, 19, 21, 22, and 37) and cingular loci (BA 23)	
		Anterograde memory	Chechko et al. (20)	Case report	fMRI (associative face-name memory task)	41yo, woman	GARA	None	During encoding, lower BOLD signal across the cingulate cortex, the superior medial frontal gyrus, the right ventrolateral and dorsolateral prefrontal cortices, the cerebellum, the bilateral parietal cortex (bilateral inferior parietal lobule, bilateral precuneus and right angular gyrus) and the right inferior and medial temporal gyri. During recognition, lower BOLD signal across the hippocampi and higher BOLD signal across the left superior frontal gyrus.	Within the first week
			Markowitsch et al. (26)	Case-report	[^15^H_2_0] PET while retrieving memories but with lack of familiarity): 3 conditions (A: resting state; B: when hearing sentences heard 1 day before; C: when hearing sentences never heard before)	30yo, man	FRA	Possible past meningitis No psychiatry comorbidity	B–C comparison: Hyperperfusion of bilateral precuneus (BA 7), bilateral parietal regions (BA 39 and BA 40), right dorsal prefrontal region and right posterior cingulate cortex Hypoperfusion of the left middle and superior temporal gyri, cortical and subcortical (putamen) motor-related regions, left thalamus and right cerebellum	6 months
After remission			Kitamura et al. (23)	Case-report	11C WAY-100635 PET (5-HT 1A receptor)	30yo, man	GARA	None	Increased 5-HT1A receptors binging of the patient in the right superior frontal cortex, right middle frontal cortex, left inferior frontal cortex in patient compared, left orbitofrontal cortex and bilateral inferior temporal cortices compared to HC after remission	5 months
After remission			Kunii et al. (24)	Case-report	SPECT	31yo, man	GARA	None	Progressively increased rCBF in prefrontal cortex during remission process	1 month
			Yasuno et al. (31)	Case-report	^[15^H_2_O]-PET (recognition of famous faces from amnestic period)	33yo, woman	GARA	None	Increased rCBF in the left temporo-occipital junction (BA19 and 37), the left anterior cingulate cortex (BA 24 and 32), the left cerebellum, the right medial and inferior prefrontal cortices (BA 6, 8 and 46), the middle temporal cortex (BA 21) and the right hippocampus (BA 35 and 36). Decreased rCBF in the right middle lateral prefrontal cortex (BA 9).	12 months
			Kikuchi et al. (35)	Case series	fMRI (face and name recognition tasks)	Case 1: 27yo, man	FRA (both cases)	None	Normalization of the pattern observed in acute phase	n.a. (partial remission)
			Mitsui et al. (40)	Case-report	SPECT	40yo, man	GARA	Active mild major depressive disorder	Normalization of the pattern observed in acute phase	6 years
			Sellal et al. (37)	Case-report	SPECT	33yo, man	FRA	None	Normalization of the pattern observed in acute phase	n.a

We identified 22 articles during the literature search. This table aims to gather the methodology and main features of each one of them. When a study could fit into two or more of the five categories (e.g., resting-state, recognition, recollection, anterograde memory, and remission), the description of the experimental design was separated into several parts.

AA, anterograde amnesia; FRA, focal retrograde amnesia; GARA, global autobiographical retrograde amnesia; LOI, loss of identity; MRI, magnetic resonnance imaging; MTR, magnetization transfer ratio; PET, positron emission tomography; SPECT, single-photon emission computed tomography.

### 3.1. Patients

Most of the patients included in the final analysis were men (*N* = 32, i.e., 65.3%). The mean age was uncertain due to the lack of data for one patient (28) and was estimated to be around 34.3 years old. Among the patients, 20 had global autobiographical retrograde amnesia (GARA) (20–31, 38, 40), 16 had focal retrograde amnesia (FRA) (32–39, 41), and 13 had loss of identity (LOI) (21, 25–28, 30, 32, 34, 38, 40). The memory impairment was not specified for the 14 patients included in the Brand et al. study (18). Symptoms were often mixed, some subjects experiencing both GARA and LOI (21, 25–28, 30, 38, 40), and others FRA and LOI (32, 34, 38).

While the majority of the patients included had no other neurological or psychiatric conditions, some of them had major depressive disorder (MDD) (25, 34, 40), one had a history of conversion disorder (25), and one had meningitis in the past (37).

### 3.2. Neuroimaging techniques

Different neuroimaging techniques were used in the studies examined, such as [^18^FDG]-positron emission tomography (PET) (18, 22, 27–29, 32), [^15^H_2_O]-PET (26, 31, 36), [^11^C-WAY-100635]-PET (23), functional magnetic resonance imaging (fMRI) (20–22, 32, 33, 35, 39), single-photon emission computed tomography (SPECT) (24, 25, 34, 36–38, 40), spectroscopy MRI (30), and magnetization transfer ratio (MTR) (30).

### 3.3. Quality of the studies

The mean overall quality of the studies included was 4.9/10, based on the scoring system detailed previously (see Table 2). 54.5% of them were controlled to HC and all cases were confirmed either by a psychiatrist or a neurologist. History of neurological or psychiatric diseases, active central nervous system conditions and active psychiatric conditions were considered respectively in 31.8%, 77.3% and 40.9% of the studies. The presence or absence of psychotropic medication at the time of the imaging procedure was explicitly stated in only five cases out of the twenty-two (22.7%).

**TABLE 2:** Assessment of the quality of the studies included in the review.

Article	Controlled to healthy subjects	Absence of neurological or psychiatric history	Absence of active central nervous system condition	Absence of active psychiatric condition	Effect of medication controlled	Diagnosis confirmed by a neurologist and/or a psychiatrist	Sufficient details for replication	Sample size	Overall score
Kitamura et al. (23)	yes	yes	yes	yes	yes	yes	yes	1	8/10
Tramoni et al. (30)	yes	yes	yes	yes	no	yes	yes	1	7/10
Hennig-Fast et al. (22)	yes	no	yes	yes	yes	yes	yes	1	7/10
Chechko et al. (20)	yes	no	yes	yes	no	yes	yes	1	6/10
Thomas-Anterion et al. (29)	yes	no	yes	yes	no	yes	yes	2	6/10
Kikuchi et al. (35)	no	no	yes	yes	yes	yes	yes	2	6/10
Sellal et al. (37)	no	yes	yes	yes	yes	yes	no	1	6/10
Markowitsch et al. (26) (Cogn. Neuropsy.)	yes	yes	yes	no	no	yes	yes	1	6/10
Magnin et al. (25)	no	yes	yes	no	yes	yes	no	1	5/10
Arzy et al. (32)	yes	no	yes	yes	no	yes	no	1	5/10
Helmes et al. (34)	no	yes	yes	no	no	yes	no	1	4/10
Kunii et al. (24)	no	no	yes	yes	no	yes	no	1	4/10
Thomas-Anterion et al. (28)	yes	no	yes	no	no	yes	no	1	4/10
Brand et al. (18)	yes	no	no	no	no	yes	no	14	4/10
Stracciari et al. (38)	no	no	yes	no	no	yes	no	13	4/10
Botzung et al. (33)	no	yes	no	no	no	yes	yes	1	4/10
Piolino et al. (27)	yes	no	no	no	no	yes	yes	1	4/10
Yang et al. (39)	no	no	yes	no	no	yes	yes	1	4/10
Glisky et al. (21)	yes	no	no	no	no	yes	yes	1	4/10
Yasuno et al. (31)	yes	no	no	no	no	yes	yes	1	4/10
Markowitsch et al. (26) (Psychiatry Res.)	no	no	yes	no	no	yes	yes	1	4/10
Mitsui et al. (40)	no	no	yes	no	no	yes	no	1	3/10

22 studies were included on the review. In this table, studies are displayed using first their overall quality and their publication date. Each one was assessed on a ten-point scale, 1 being of very low quality and 10 being of very high quality. Every positive answer to one of our criteria added one point. Studies with 15 patients or more were rewarded 3 points; the ones between 5 and 14 included gave 2 points, and studies with less than 5 patients were only given 1 point.

### 3.4. Brain areas and systems

#### 3.4.1. The frontal regions

At rest, patients with DA demonstrated a decreased [^18^FDG]-PET metabolism in the right inferior lateral prefrontal cortex (PFC) compared to HC in the Brand et al. study (18). In other studies compared to HC, metabolism was decreased in the right ventral frontal gyrus (27) and increased in the left inferior frontal cortex (32). At rest, the regional cerebral blood flow (rCBF) was decreased in the bilateral frontal superior and orbitofrontal gyri (25), the left frontal pole (34, 38) and the right inferior frontal areas (36, 38) but none of these reports were controlled. Finally, a decreased MTR and a lower MAA/(Cho + Cr) ratio appeared in the right PFC in a magnetic resonance spectroscopy study (30) compared to HC.

During recognition tasks, patients had an increase in their Blood-Oxygen-Level Dependent (BOLD) signal in the left orbitofrontal region (20), the left inferolateral gyrus (22) compared to controls. The BOLD signal was also decreased in the bilateral dorsolateral PFC, the left ventromedial and ventrolateral PFC (35) within the same patients during face-name recognition tasks. Recognition was also associated with an increased rCBF compared to HC in the right medial PFC (31).

While recollecting memories from the past, patients with DA had a lower BOLD signal in the left dorsolateral PFC and the right middle lateral frontal gyrus (33).

During the encoding of new memories, they presented a lower BOLD signal in the bilateral superior medial PFC and the right ventrolateral and dorsolateral PFC (20), as well as an increased rCBF in the right dorsal PFC (36).

Finally, after remission, patients exhibited increased 5HT binding in the right superior and middle frontal cortex, the left inferior frontal cortex and the left orbitofrontal cortex compared to the amnesic state in a within-subject analysis (23). Several case reports described a normalization of the patterns observed (31, 35), with a progressive increase in PFC perfusion during the process (24).

#### 3.4.2. The temporal lobe

At rest, patients with DA had a decreased metabolism in the right temporo-mesial region (22, 29, 37), the left temporo-polar cortex (28, 32), the left hippocampus and para-hippocampus (28), and the left fusiform gyrus (28). Their rCBF was decreased in the temporal lobe (25, 34, 40). These patterns normalized upon resolution of the symptoms.

During the recognition process, patients showed a higher BOLD signal in the bilateral middle (21), the left inferior and the right mesial temporal gyri (22). The BOLD signal was higher in the bilateral fusiform gyri as well (22), with left predominance (20). A lower BOLD signal was also observed in the para-hippocampi (39) and hippocampi (20, 33, 35, 39) and normalized after remission (35). The rBCF was decreased in the right temporal lobe (26, 31). When patients tried to recollect memories, their left para-hippocampus failed to activate and showed a decreased BOLD signal (33). During encoding, patients with DA had a lower BOLD signal in the right inferior and medial temporal gyri (20), associated with a decreased rCBF in the left middle and superior temporal lobe (36). Finally, at remission, besides several normalizations of the pattern observed (31, 35, 37), patients also had increased 5HT binding in the bilateral inferior temporal cortex (23).

#### 3.4.3. The limbic system

In a resting state, patients with DA had a decreased metabolism in the left insula (28), and the left amygdala (28). They also showed a decreased rCBF in the caudate nucleus (25).

When trying to recognize memories from their amnestic period, a higher BOLD signal was observed in the amygdalo-hippocampal complexes (20) and the left anterior cingulate cortex (ACC) (22), whereas a lower BOLD signal was observed in the striatum (21) and the bilateral amygdalae and insulae (39). Patients’ rCBF was decreased in the right insula (26, 31) and increased in the left ACC, the left caudate, the left putamen and the right anterior medial regions, including the amygdala (which normalized after the resolution of the symptoms) (31). During the recollection of memories, patients with DA had decreased activation in the left ACC and greater activation in the posterior cingulate cortex (PCC) (33). Finally, during anterograde memory tasks, patients with DA had an decreased BOLD signal in the cingulate cortex (20), associated with a lower rCBF in the right PCC (36). Their rCBF was decreased in the left putamen and thalamus (36).

#### 3.4.4. Other brain areas

Patients with DA had altered parietal region functioning. At rest, they showed an increased metabolism in the bilateral posterior parietal cortex, with left predominance (32). When they tried to recognize memories from the forgotten period, case reports showed an increased BOLD signal in the bilateral posterior parietal cortex (32), the precuneus (20) [with an increased rCBF (36)] and the bilateral inferior parietal lobule (20). One case report also reported a lower rCBF in the right inferior parietal cortex, which normalized at remission (31). Finally, during the encoding process, patients had a lower BOLD signal in the bilateral parietal cortex including the inferior lobule, the precuneus and the right angular gyri (20), associated with a higher rCBF in these regions (36).

In the occipital lobe, during the recognition process, patients showed a higher BOLD signal in the bilateral middle occipital gyri (20), the bilateral inferior occipital gyri (22), the bilateral cuneus (20), and the left lingual gyrus (22).

Cerebellum function was altered, too, with a decreased rCBF in the right hemisphere at rest (34, 36) and a lower BOLD signal during encoding (20).

Finally, when patients with DA tried to recollect memories from their amnestic past, one case report showed an increased BOLD signal in the temporo-parieto-occipital junction (33).

#### 3.4.5. Normal results

Some studies (26, 35, 38) reported normal results for neuroimaging procedure that were performed while patients were amnestic. rCBF was considered normal in seven patients (two of them from Kikuchi’s study and five in Stracciari’s one) (35, 38). However, none of these normal results was controlled to healthy subjects. In a case report, Markowitsch compared the rCBF of one patient to a group of seven HC and failed to find any difference (26).

### 3.5. Pure GARA vs. pure FRA

Seven case reports focused on patients with pure GARA (20, 22–24, 29, 31, 40) and six on patients with pure FRA (33, 35–39). We tried to highlight the common points between the two clinical states. The only shared pattern between pure GARA and pure FRA was decreased activity in the temporal pole at rest compared to controls (22, 29, 40). No common point was found in the recognition collection, noticeably with divergent results using fMRI techniques. Due to the difference in the imaging methods used for the encoding and recollection process evaluations, comparison was not possible (see Table 1).

### 3.6. Acute vs. chronic state

We finally tried to bring to the light the putative differences between acute and chronic state in patients with DA. We could not find common points between the acute and chronic state at rest. During recognition, both acute and chronic patients had a decreased rCBF in their right temporal regions, including the amygdalar and hippocampal structures (31, 36).

The comparison of the two states was not possible for encoding (no comparable imaging technique) or recollecting (lack of data for acute state).

## 4. Discussion

Numerous reports studied the neural mechanisms underlying the neural correlates of DA. Despite their heterogeneity, these reports predominantly showed a functional modification in the frontal areas, the limbic structures and the temporal lobe. Marginally, some reports showed abnormalities in the posterior and inferior parietal cortex, the occipital cortex and the cerebellum. Four of the six studies with another brain imaging technique after remission showed a normalization of the patterns observed when patients were amnesic. In the frontal regions of the brain, at rest, patients with DA mostly presented hypoactivity of the right inferolateral PFC, the right frontal gyrus and the bilateral frontobasal, frontomesial and orbitofrontal regions compared to HC (18, 27, 32). When performing a memory task, the patients with DA had greater activation in the frontal areas of their brains during recognition tasks, while their left dorsolateral PFC and right middle lateral frontal cortex failed to activate correctly during recollection. However, those results rely on uncontrolled studies and replication are need. Besides the frontal abnormalities described above, at rest, patients with DA showed diminished activation of their temporal areas compared to HC, especially in the left hemisphere (22, 28, 29, 32). Moreover, when they tried to recognize or recollect memories from their amnestic past, they failed to activate their bilateral hippocampi and para-hippocampi compared to HC (20), leading to lower BOLD signal in the studies reviewed. Finally, patients with DA had decreased activation of their limbic system (noticeably in the left insula, amygdala, and putamen) at rest compared to controls (28) but demonstrated broad abnormalities during memory tasks. They showed hyperactivity of their amygdalo-hippocampal complexes and their ACC during the recognition process compared to HC (20–22), while these regions were deactivated when the patients tried to recollect memories. The only common point between pure GARA and pure FRA patients was a decreased activity in the temporal lobe at rest. The comparison between acute and chronic states only highlighted a shared pattern of decreased rCBF through the temporal lobe (encompassing hippocampi and amygdalar structures). These data seem to favor an involvement of the temporal lobe as a core node of DA, independently of the duration of the symptoms or the time span encompassed by the amnestic state. Noticeably, three studies gathering eight patients reported normal SPECT examinations, but the results were controlled to HC in only one subject.

**FIGURE 2:**
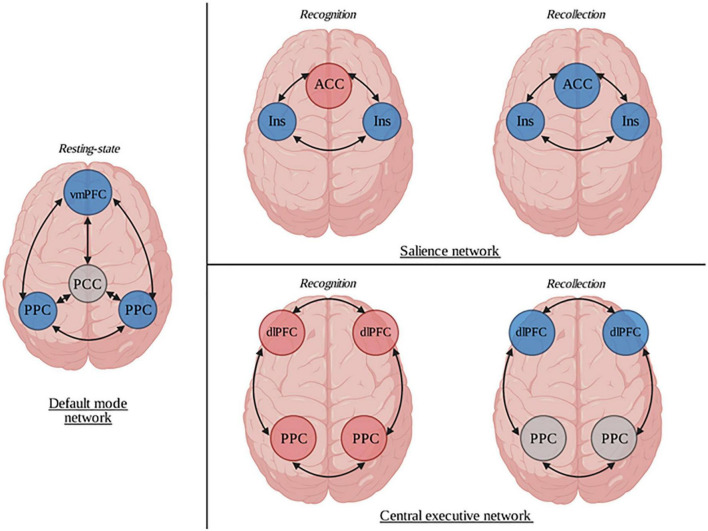
Representation of the neural correlates of dissociative amnesia (DA). Neuroimaging studies of DA show that patients had several differences in their brain functioning compared to healthy subjects. In a resting state condition, through the default mode network, they had a decreased activation of the ventromedial prefrontal cortex and the bilateral posterior parietal cortices. Abnormal patterns were also identified during recognition and recollection tasks in both salience and central executive networks. ACC, anterior cingular cortex; dlPFC, dorsolateral prefrontal cortex; Ins, Insula; PCC, posterior cingular cortex; vmPFC, ventromedial prefrontal cortex. Blue circles represent hypoactivations. Red circles represent hyperactivations. Gray circles represent normal functioning.

Three core brain networks that play a key role in the coordination of cognitive, affective and interpersonal processing have been identified: the default mode network (DMN), the central executive network (CEN), and the salience network (SN). Patients with DA show large anatomically dispersed abnormalities in each one of these three networks (see Figure 2). The DMN is constituted of functionally correlated brain areas (PCC, ventromedial PFC, medial, lateral, and inferior parietal cortex) (42). Among other functions, it is implicated in the interplay between emotional processing and cognition (43). Its increased function is demonstrated in self-related mental activity (44). Patients with DA had decreased activation of their ventromedial PFC (25, 27) and their posterior parietal cortex (32) at rest compared to HC. CEN, on the other hand, is a network antagonist to the DMN; this network is anchored in the bilateral dorsolateral PFC and the lateral posterior parietal cortex. It tends to activate in emotional and higher-order mental states (45). In the studies reviewed, the activation of the CEN was different depending on whether the patient was intensively recollecting memories or only recognizing them. In recognition, the BOLD signals observed in their dorsolateral PFC and their lateral posterior parietal cortex were higher than in HC (32, 35), while the dorsolateral PFC failed to activate during recollection (33). Lastly, the dynamic switch between the arousal of the DMN or the CEN is dependent upon the SN. More precisely, saliency detection has prominent consequences on how internally- (DMN) and externally-directed (CEN) cognitions are processed by one’s conscience (46). The SN is a large-scale network whose two major nodes are the dorsal part of the ACC and the anterior insulae, but it also includes the amygdala, the ventral striatum and the ventral tegmental area. Two patterns of SN activation–depending on the memory task performed (recognition or recollection)–emerge from the studies included. During recognition, patients with DA had a diminished rCBF in their insulae (36), and an increased BOLD signal and rCBF were observed in the ACC (22, 31) and the amygdala (20, 31) compared to controls, while both their insulae and ACC failed to activate during recollection (33, 39). Based on these observations, a network-centered approach may be useful to better understand the neural basis of DA and to gather the sparse results of the scientific reports previously reported.

Approximately three out of four DA are related to an identified traumatic event (4), establishing a strong link between stress and this disorder. Memory suppression is a coping strategy that helps individuals to suppress emotional memories through two time-differentiated procedures (47): the first implies the progressive deactivation of the right inferior frontal gyrus, the pulvinar and the fusiform gyrus, while the second is linked to the decreased activity of the amygdalo-hippocampal complex, which is anti-correlated to the right medial frontal gyrus’ activation over time. Patients with post-traumatic stress disorder (PTSD) have increased activation of their dorsolateral PFC (48) and increased functional connectivity between the inferior frontal gyrus and the para-hippocampus during experimental memory suppression tasks (49) compared to controls. Phenomenologically speaking, while PTSD could reflect the failure of the memory suppression system to remove unwanted recollections from the patient’s mind, DA might, on the contrary, be considered an involuntary overactivation of this system, eliminating the ability to retrieve any autobiographical episode (see Table 3). Several studies documented a dysfunction in brain regions involved in memory suppression in DA, such as the right dorsolateral PFC (35), the right inferior lateral frontal cortex (18, 36), the hippocampi and the para-hippocampal regions (20, 31). One study used a think/no-think paradigm to study the involvement of memory suppression in DA (30). However, because of its design (a single case experiment), it is difficult to draw any firm conclusion about the involvement of the memory suppression system in DA.

**TABLE 3:** Common point and contrasts between dissociative amnesia and post-traumatic stress disorder.

	Dissociative amnesia	Post-traumatic stress disorder
Core clinical feature	Inability to recall numerous autobiographical memories	Inability to suppress the recollection of one episodic memory
Exposure to a traumatic event	Around 75%	Mandatory
Neural network-based interpretation	Over-activation of the memory suppression system	Ineffective memory suppression system
Neuroimaging correlates	• Increased activity in the amygdalar-hippocampal complex	•Increased activation of the dorsolateral prefrontal cortex •Increased functional connectivity between the inferior frontal gyrus and the para-hippocampus

Dissociative states are still poorly understood. The two other main clinical entities alongside DA in the dissociative disorders chapter in the DSM are dissociative identity disorders and depersonalization/derealization disorders. Patients with dissociative identity disorder have smaller hippocampal (17, 50) and amygdalar (17) volumes than controls, an decreased rCBF in the bilateral orbitofrontal cortex and an increased rCBF in the median and superior frontal regions and the occipital areas bilaterally (51). Patients with depersonalization and derealization disorders have a lower fractional anisotropy than HC within the right temporo-parietal junction and the left temporal lobe (52). Their right ventrolateral PFC seems to be hyperactivated in depersonalization disorder, with a leading role of this structure in the “top-down” inhibition of emotional responses (53). The constellation of brain areas linked to dissociative experiences previously identified in the literature overlaps with the brain regions identified as dysfunctional in patients with DA. Noticeably, this includes brain areas involved in the DMN (bilateral orbitofrontal cortex, posterior parietal cortex) and the hippocampi, which are of interest in DA, as described above.

Yet, despite some converging results, several limitations appeared. First, the population included might not be representative of DA patients. Indeed, while we expected a 1:1 sex ratio based on the epidemiology, most of the subjects included in the studies were men. Although this may be linked to a probable sampling bias due to the limited number of patients included in the review, it is possible that sex plays a role in the results. Secondly, patients included in the review were very heterogeneous in term of clinical features (ranging from GARA to FRA and sometimes LOI) and comorbidities. It is obvious that the actual presence of an active comorbidity such as depression or functional neurological disorder may have influenced the results of the studies. We tried to compare GARA and FRA patients but failed to draw any conclusion because of the disparate imaging techniques employed to assess the brain modifications, and we were not able to analyze imaging data based on a LOI grouping because of the heterogeneity of such a thing. This heterogeneity adds another constraint to the generalization of these data. The same comment applies to the delay between the onset of symptoms and the imaging procedure. It is worth noting that over time, the changes observed in amnestic patients may be different because of mechanisms such as neural plasticity, re-learning their own biographical information or even social, familial or behavioral reinforcers. We also tried to compare the data on chronic and acute states, but once again, the comparison was made difficult by the numerous paradigms employed. Finally, it is possible that the sole fact of observing the phenomenon might influence the results, known as the Hawthorne effect. In most cases, controlling the results to those of HC might be helpful to limit this effect, but only one of the studies included in the review presented data compared to a control population. Our review emphasized the lack of prospective controlled studies in the field of neuroimaging in DA. The overall quality of the 22 studies included was poor and some of the most obvious confounding factors were not often considered such as history or active neurological or psychiatric condition, or medication for example (see Table 2). Additionally, the methods varied greatly, both in the neuroimaging technique employed and the tasks performed (see Table 1). It is thus exceedingly difficult to draw any firm conclusions. Surprisingly, we failed to find a single study using a resting-state fMRI paradigm. Resting-state fMRI is a promising tool to study brain function, and further studies employing this technique seem mandatory in the years to come to help better understand the pathophysiology of DA.

Progress made in the field of non-invasive brain stimulation techniques were very important over the past decades. It is now commonly used to treat some classic psychiatric conditions such depressive disorders (54) or refractory hallucinations (55). Moreover, recent experimental approaches also demonstrated their efficacy in neurological-like psychiatric disorders such as functional weakness (56) and functional motor disorders (57). Techniques like repetitive transcranial magnetic stimulation or transcranial direct current stimulation have also been used to enhance cognitive functioning, and a recent review revealed that non-invasive brain stimulation had a small but significant pre-cognitive effect on attention and working memory (58). A better description of the brain areas involved in DA may be of use to determine new targets for non-invasive brain stimulations and eventually be a future lead in the treatment of this neuropsychiatric condition.

## 5. Conclusion

Dissociative amnesia is an uncommon neuropsychiatric condition. Its pathophysiology is unclear, but our review in the field of neuroimaging studies suggests abnormal functioning of the DMN at rest and of the SN and the CEN when patients attempt to retrieve memories. The underlying mechanism might involve an overly activated memory suppression system, but further studies are needed to confirm this theory. The scientific literature about DA lacks methodologically strong studies, and despite numerous reports published in the field of neuroimaging in DA, only one of these publications is a prospective and controlled study, the remaining articles being, for the most, case reports. Moreover, most of the studies included used molecular imaging and task-performing fMRI. More recent approaches in the field of neuroimaging like resting-state fMRI could be of use to define more precisely the discrepancies among brain networks in future experiments. As very few forms of care management have scientifically shown their efficacy for DA yet, the unveiling of these *in vivo* mechanism using functional imaging may help scientists to define novel targets for neuromodulation treatments in this neuropsychiatric disorder.

## Author contributions

ST, AY, BL, PP, and JP contributed to the conception of the study and made significant participation upon manuscript draft. ST, AY, and JP acquired the data (reviewing process). All authors contributed to the article and approved the submitted version.
